# K21-Antigen: A Molecule Shared by the Microenvironments of the Human Thymus and Germinal Centers

**DOI:** 10.1155/1998/29340

**Published:** 1998

**Authors:** Nesrina Imami, Heather M. Ladyman, Bjarne Vincents, Abdulhamid Al-Tubuly, Jona Freysdóttir, Moditi L. Sedibane, David A. Taylor-Fishwick, Brian M.J. Foxwell, Mary A. Ritter

**Affiliations:** 1 Department of Immunology Imperial College School of Medicine Hammersmith Hospital DuCane Road London W12 ONN UK; 2 The Kennedy Institute of Rheumatology and Charing Cross Sunley Research Centre Hammersmith London W6 8LW UK

**Keywords:** Follicular dendritic cell, germinal center, lymphocyte development, thymic epithelium, thymic microenvironment, thymus

## Abstract

The mouse IgG1 monoclonal antibody (mAb) K21 recognizes a 230-kD molecule (K21-Ag) on Hassall's corpuscles in the human thymus. This mAb also stains cultured thymic epithelial cells as well as other epithelial cell lines, revealing a predominant intracellular localization. Further analysis with mAb K21 on other lymphoid tissues showed that it also stains cells within the germinal centers of human tonsils, both lymphoid (B) cells and some with the appearance of follicular dendritic cells. Double immunostaining of tonsil sections shows that K21-Ag is not expressed by T cells, whereas staining with anti-CD22 and -CD23 mAb revealed some doublepositive cells. A subpopulation of the lymphoid cells express the K21-Ag much more strongly. This K21^++^/CD23^++^ subpopulation of cells is localized in the apical light zone of germinal centers, suggesting that K21-Ag may be an important marker for the selected centrocytes within germinal centers and may play a role in B-cell selection and/or development of B-cell memory. Flow cytometric analysis showed that K21-Ag is expressed on the surface of a very low percentage of thymocytes, tonsillar lymphocytes, and peripheral blood mononuclear cells. Analysis of purified/separated tonsillar T and B lymphocytes showed that T cells do not express the K21-Ag; in contrast, B cells express low levels of the K21-Ag, and this together with CD23 is upregulated after mitogenic stimulation. Our data therefore raise the possibility that the K2l- Ag may play a role in B-lymphocyte activation/selection.


*Developmental Immunology,* 1998, Vol. 6, pp. 41–52
Reprints available directly from the publisher
Photocopying permitted by license only

© 1998 OPA (Overseas Publishers Association)
N.V. Published by license under the
Harwood Academic Publishers imprint,
part of the Gordon and Breach Publishing Group
Printed in Malaysia

### K21-Antigen: A Molecule Shared by the Microenvironments of the Human Thymus and Germinal Centers

NESRINA IMAMI[a]*, HEATHER M. LADYMAN[a], BJARNE VINCENTS[a], ABDULHAMID AL-TUBULY[a],, JONA FREYSDÓTTIR[a], MODITI L. SEDIBANE[a], DAVID A. TAYLOR-FISHWICK[b], BRIAN M.J. FOXWELL[b] and MARY A. RITTER[a]

[a]*Department of Immunology, Imperial College School of Medicine, Hammersmith Hospital, DuCane Road, London W12 0NN, United Kingdom*; [b]*The Kennedy Institute of Rheumatology and Charing Cross Sunley Research Centre, Hammersmith, London W6 8LW, United Kingdom*

*(Received 20 October 1996; Accepted 9 May 1997)*

The mouse IgG1 monoclonal antibody (mAb) K21 recognizes a 230-kD molecule (K21-Ag) on Hassall's corpuscles in the human thymus. This mAb also stains cultured thymic epithelial cells as well as other epithelial cell lines, revealing a predominant intracellular localization. Further analysis with mAb K21 on other lymphoid tissues showed that it also stains cells within the germinal centers of human tonsils, both lymphoid (B) cells and some with the appearance of follicular dendritic cells. Double immunostaining of tonsil sections shows that K21-Ag is not expressed by'T cells, whereas staining with anti-CD22 and -CD23 mAb revealed some double-positive cells. A subpopulation of the lymphoid cells express the K21-Ag much more strongly. This $K21^{++}/CD23^{++}$ subpopulation of cells is localized in the apical light zone of germinal centers, suggesting that K21-Ag may be an important marker for the selected centrocytes within germinal centers and may play a role in B-cell selection and/or development of B-cell memory. Flow cytometric analysis showed that K21-Ag is expressed on the surface of a very low percentage of thymocytes, tonsillar lymphocytes, and peripheral blood mononuclear cells. Analysis of purified/separated tonsillar T and B lymphocytes showed that T cells do not express the K21-Ag; in contrast, B cells express low levels of the K21-Ag, and this together with CD23 is upregulated after mitogenic stimulation. Our data therefore raise the possibility that the K21-Ag may play a role in B-lymphocyte activation/selection.

*Keywords:* Follicular dendritic cell, germinal center, lymphocyte development, thymic epithelium, thymic microenvironment, thymus

### INTRODUCTION

Monoclonal antibodies (mAbs) have provided valuable tools with which to study anatomical and functional niches within the thymic microenvironment (Boyd et al., 1993; Ritter and Boyd, 1993). Several of these mAbs, raised in our laboratory to both human and mouse thymic epithelial cells, have

*Corresponding author.

been shown to detect antigens shared by thymic epithelium and leukocytes (DeMaagd et al., 1985; Kampinga et al., 1989; Imami et al., 1992). One such molecule, gp200-MR6, which is expressed by human thymic cortical epithelium, is thought to be functionally associated with the human interleukin-4 receptor (IL-4R; Larché et al., 1988; Imami et al., 1994; Al-Tubuly et al., 1996). These data, together with our observations that the phenotype of thymic epithelium can be modulated by IL-4 suggested that lymphocyte-derived IL-4 might play a role in maintaining the thymic microenvironment (Freysdóttir and Ritter, 1998). In order to further analyze IL-4R structure and function on thymic epithelium, we attempted to generate mAbs to the human IL-4R following immunization of (CBA/BALB/c)$F_1$ mice with keyhole limpet hemocyanin (KLH)-coupled synthetic peptides based on the published cDNA sequence of the 140-kD chain of the IL-4R (CD124; Galizzi et al., 1990). After fusion and subsequent cloning, several mAbs were identified that were reactive with thymic epithelial cells and lymphocytes, although their distribution did not resemble that of the IL-4R, as revealed by fluoresceinated IL-4 (Mat et al., 1991).

We have now analyzed these mAbs further and show that one of them, mAb K21, which labels thymic Hassall's corpuscles and surrounding patches of medullary epithelium, also recognizes tonsillar epithelium and germinal center cells—both lymphoid cells and some with the appearance of follicular dendritic cells. Further studies with two-color immunohistochemistry, flow cytometry, Western blotting, and mitogen activation have been undertaken to determine the nature of the K21-Ag, and the lineage and functional status of the cells that express it. These studies should help to elucidate the role of the thymic epithelium and its similarity with other lymphoid microenvironments.

### RESULTS

### Distribution, Expression, and Biochemical Analysis of the K21-Ag

MAb K21 was raised against a cocktail of KLH-coupled synthetic peptides of the IL-4R (Galizzi et al., 1990). It was screened by ELISA using individual peptides (coupled to BSA) and showed strong reactivity with WSXWS, but was not reactive with other peptides. As this motif is a functional part of all cytokine receptors within the haematopoietin cytokine receptor superfamily, including IL-2R$\beta$ (CD122), IL-4R (CD124), IL-7R (CD127), and the common $\gamma$ chain ($\gamma$c), we pursued with analysis of this mAb (Bazan, 1990; Miyajima et al., 1992; Sugamura et al., 1996). Additionally, the mAb K21 was screened on human thymus and was shown to strongly label some Hassall's corpuscles and occasionally the associated medullary epithelium (cluster of thymic epithelial staining; CTES group V). The K21$^+$ Hassall's corpuscles are generally those that are medium to large in size (Figure 1). Isotyping showed it to be an IgG1 mAb.

Western blotting analysis, using freshly prepared lysates from frozen tonsillar tissue, revealed that K21-Ag comprised one band with an apparent molecular weight of approximately 230 kD (Figure 2). Western blotting carried out under reducing conditions, using either 2-Mercaptoethanol (2-ME) or Dithiothreitol (DTT), destroyed the epitope recognized by mAb K21. Flow cytometric analysis of thymic epithelial cells from primary cultures (Figure 3) shows that most of K21-Ag is expressed internally and that this expression increases over 5 to 8 days in culture medium. Neither IFN$\gamma$ nor IL-4 had any effect on the expression of this molecule (data not shown). In addition, indirect immunofluorescence staining and flow cytometric analysis of the human epithelial cell line (HT29) showed that this molecule is also expressed internally in other epithelial cell lineages, whereas surface molecules are also present on a small percentage of cells (2-5%; Figure 4). Indirect immunoperoxidase staining of these cells in culture confirmed the predominant intracellular localization of the K21-Ag (Figure 5).

When tested on other lymphoid tissue, mAb K21 revealed staining of human tonsils. Single immunoperoxidase staining of human tonsil sections showed that this mAb stains the stratified squamous tonsillar epithelium and cell populations within the germinal centers of the follicles where both the B cells

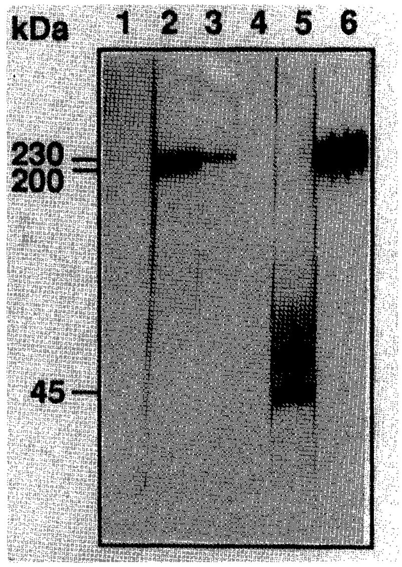

FIGURE 2 Western blotting analysis. Frozen tonsil tissue lysates were subjected to SDS-10%PAGE and immunoblotting. Negative control (lane 1); gp200-MR6 (200 kD, lane 2); K21-Ag (230 kD, lane 3); CD4 (negative, lane 4); CD23 (45 kD, lane 5); and CD45 (220 kD, lane 6). Under nonreducing conditions, the K21-Ag has an apparent molecular weight of 230 kD.

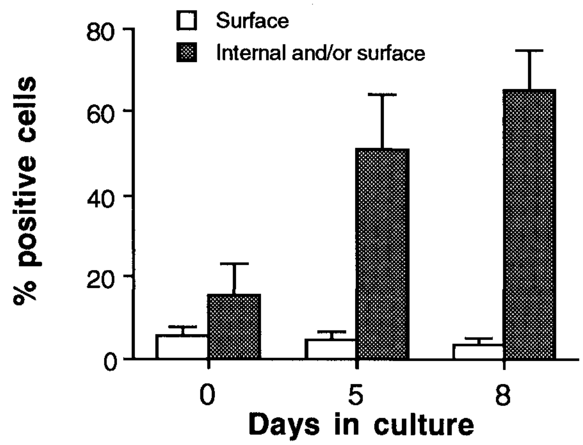

FIGURE 3 Flow cytometric analysis of either surface or internal and/or surface expression of K21-Ag on cultured human thymic epithelial cells. Cells were isolated and cultured for 0-8 days in supplemented culture medium only. Results are expressed as mean percentage of stained cells ± standard deviation of three experiments.

lymphocytes showed that T cells do not express the K21-Ag, whereas ~14% of B cells are K21-Ag-positive (Table I). Flow cytometric analysis of separated tonsillar B lymphocytes after 24-hr mitogen stimulation (lipopolysaccharide, LPS) raised the percentage of K21-Ag-positive cells from 14% to 62% (total percentage of B cells was 80%). Therefore, it appears that K21-Ag expression is upregulated upon B-cell activation.

### DISCUSSION

The aim of this study was to raise mAbs to the IL-4R as part of the project to explore the role of cytokine/cytokine receptor interactions between the thymic stromal microenvironment and the maturing T lymphocytes, since it has been shown that interactions such as those occurring between IL-4/IL-4R and IL-7/IL-7R are important in development of both thymocytes and the thymic epithelial component (Ritter and Boyd, 1993; Peschon et al., 1994; Zlotnik and Moore, 1995; Freysdóttir and Ritter, 1998). Although we immunized with known peptides of the IL-4R, after the fusion, selection, and subsequent cloning, none of the mAbs showed recognition of the IL-4R. However, some of them showed reactivity with thymocytes and/or the thymic epithelial cells. K21, which recognized Hassall's corpuscles and associated medullary epithelium, was selected for further study.

It is not clear why the methodology used failed to generate mAbs to the IL-4R. Mice were immunized with a mixture of five different synthetic peptides based on the sequence of the human IL-4R. Peptides were selected for their potential immunogenicity and exposure on the surface of the native molecule, and were coupled to KLH to provide a highly immunogenic carrier. The failure to generate anti-IL-4R mAb may result from close conservation of sequence between mouse and man; alternatively, the three-dimensional structure of the peptide-carrier complexes may differ from that of the equivalent sequence in the native molecule.

The generation of non-IL-4R mAb was not unexpected since similar findings have been observed previously in our laboratory as well as by other investigators (Sharif et al., 1990; Freysdóttir et al., manuscript in preparation). One possibility is that we have selected auto-antibody producing cells that cross-react between species. Thus, human anti-GlcNAc antibodies cross-react with cytokeratin from human skin (Shikhman and Cunningham, 1994), although the 230-kD K21-Ag is too large to be a keratin (Laster et al., 1986). Moreover, the mAb TOTO-1 generated from a patient with systemic lupus erythematosus (SLE) has been shown to be reactive with Hassall's corpuscles, indicating the presence of autoreactive antibodies with specificity for thymic epithelium (Numasaki et al., 1995).

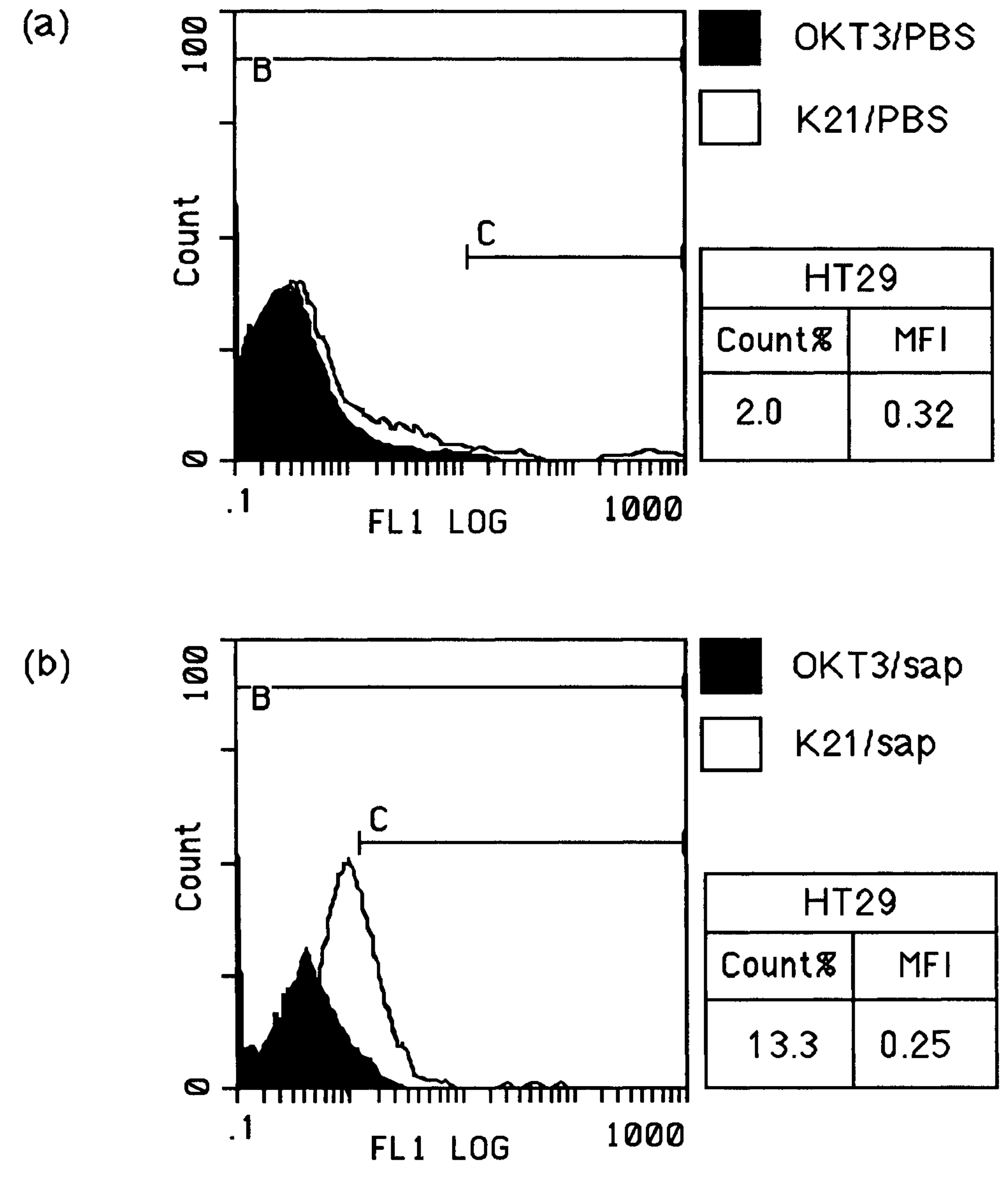

FIGURE 4 K21-Ag expression on HT29 human epithelial cell line analyzed by flow cytometry: (a) surface, and (b) internal and/or surface expression levels. Isotype-matched control mAb (OKT3) was used as a negative control (<2.0% in both cases).

TABLE I Flow Cytometric Analysis Using mAb K21 on Separated Tonsillar T Cells (E-Rosetted) and B Cells (Nylon Wool Bound)[a]

| | % stained cells | |
|---|---|---|
| | E-rosetted (T) cells | Nylon wool bound (B) cells |
| CD20 | 9 | 91 |
| CD19 | 8 | 96 |
| CD3 | 86 | 5 |
| CD7 | 85 | 4 |
| CD14 | 1 | 3 |
| CD68 | 3 | 1 |
| K21-Ag | 3 | 14 |
| Negative control | 2 | 3 |

[a]Experiments were carried out three times and a representative experiment is shown.

HT29 cells in culture confirmed the predominant intracellular localization of this antigen. Whether the K21-Ag functions intracellularly or whether the intracellular molecules provide a pool from which molecules can rapidly be recruited to the cell surface is not known.

MAb K21 also stains tonsillar epithelial cells. This fits well with the data of Perry and Slipka (1993), who studied the formation of the tonsillar corpuscles (epithelial structures found in the vicinity of the crypts) and showed that these structures closely resemble thymic Hassall's corpuscles (a phenomenon explained by endodermal embryological origin of the primordia of both organs; Perry and Slipka, 1993). K21 also labels patches of cells that resemble follicular dendritic cells in the tonsillar germinal centers, and is expressed at low levels on tonsillar B cells (identified by their location and dual staining with B-lineage markers). K21-positive cells in the T-cell area are CD5-negative, thus indicating that T cells do not bear this antigen and that the scattered positive cells are likely to be B cells. The presence of K21-Ag in/on follicular dendritic cells indicates that for both germinal centers and thymus (Hassall's corpuscles), the K21-Ag seems to be present on elements of the microenvironment where lymphocyte selection and apoptosis occur (Gray, 1993; Douek and Altmann, manuscript in preparation), suggesting a role for K21-Ag in the interaction of the selected lymphocytes with their microenvironment.

Further flow cytometric and functional analysis showed that a subpopulation of B cells is K21-Ag-positive and that this is expanded on activation. Since activation is associated with the events of lymphocyte selection, this raises the possibility that K21-Ag in the tonsil is present on both the selecting microenvironment and on the selected cells, paralleling data obtained with CD23 (Liu et al., 1991; MacLennan et al., 1992; Gray, 1993; MacLennan, 1994). A similar situation may exist in the thymus since B lymphocytes have been shown to be present in the normal human thymic medulla; these are concentrated around Hassall's corpuscles and often have rosettes of thymocytes around them (Spencer et al., 1992).

Various disorders, including infections, therapeutic treatments, irradiation, and immune deficiencies, are associated with thymic involution and loss of medullary epithelium, including/excluding loss of Hassall's corpuscles. In HIV-related disease, in the pediatric population of of the most common disorders is thymic dysinvolution with loss of Hassall's corpuscles (Mishalani et al., 1995). In contrast, in adults with early stage of AIDS, the adipose-involuted thymus shows persistence of many Hassall's corpuscles together with lymphoid follicular hyperplasia (Prevot et al., 1992). These differences might reflect differences in the lymphocyte populations involved in the two disease situations since maintenance of medullary epithelium is known to be influenced by the surrounding thymocytes (Ritter and Boyd, 1993). Thus, loss of mature medullary thymocytes either due to cyclosporin A treatment or in T-cell-deficient mice leads to loss of the thymic medullary epithelium (including Hassall's corpuscles) (Kanariou et al., 1989; van Ewijk, 1991; Palmer et al., 1993).

The mAb K21 therefore recognizes a novel 230-kD molecule expressed by microenvironmental cells of thymus and tonsil that play an important role in lymphocyte selection. Investigations to define more fully the structure and function of this molecule and to determine its role, if any, in lymphocyte-selection events are currently in progress.

### MATERIALS AND METHODS

### Source of Tissues, Cells, and Cell Lines

#### Human thymus and tonsil tissue and tissue sections

Thymus samples were obtained from children undergoing cardiac surgery (Great Ormond Street Hospital for Sick Children, London) and freshly excised tonsils from children that had undergone tonsillectomy (ENT Clinic, St. Mary's Hospital, London). Six-micron tissue sections cut from liquid nitrogen frozen blocks were air dried for 24 hr, fixed in acetone (BDH, Poole, UK) for 10 min, and either used immediately or stored at −20°C until used.

#### Thymocytes, tonsillar lymphocytes, and PBMC

Fresh thymus or tonsil tissue was thoroughly teased to release the lymphocytes, samples were allowed to sediment for 10 min, and the lymphocyte-containing supernatant was collected and washed three times in HEPES (20 mM) buffered RPMI 1640 (ICN Flow, Thame, UK). The recovered cell suspension was then centrifuged over Ficoll-Hypaque (688 g, 20 min; Pharmacia Biotech, St. Alban's, UK). Mononuclear cells were collected from the interface and washed twice in HEPES/RPMI. Medium containing 50 $\mu$g/ml gentamycin was used for tonsils. Peripheral blood from healthy donors was collected into heparinized 30-ml universal containers (Bibby Sterilin, Stone, UK) containing 100 U of preservative-free heparin (Leo Laboratories, Princes Risborough, UK). PBMC were isolated by density-gradient centrifugation using Ficoll-Hypaque as described before.

#### Fresh stromal cells and human thymic epithelial cell cultures

The remaining thymic stroma (sedimenting fragments) were processed by digestion in collagenase and epithelial cells were further isolated and cultured as described by Freysdóttir and Ritter (1998). Cells were cultured for 8 days in either medium only, IFN$\gamma$ (100 U/ml) or IL-4 (25 U/ml) (both Genzyme, West Malling, UK), harvested on days 0, 5, and 8, and analyzed by flow cytometry as described in what follows.

#### Human epithelial cell line, HT29

This is a well-differentiated colonic carcinoma cell line (American Type Culture Collection, ATCC, Rockville, MD; Fough and Trempe, 1975). Cells were cultured in RPMI 1640 supplemented with 10% fetal calf serum (FCS), L-glutamine (2 mM), sodium bicarbonate (2 g/l), penicillin (100 IU/ml), and streptomycin (100 $\mu$g/ml) (all ICN Flow). HT29 epithelial cells were grown in glass slide flasks (Nunc, Roskilde, Denmark) until confluent, washed extensively in phosphate-buffered saline (PBS), fixed in acetone for 10 min, and the immunoperoxidase staining was performed as described in what follows.

### Source of Antibodies

K21 is an $IgG_1$ murine mAb, reactive with Hassall's corpuscles of the human thymic epithelium, raised following i.p. immunization of $(CBA/BALB/c)F_1$ mice with synthetic peptides (MKVLQXPTCVSDY, KPSXHVKPR, SXNDPADFRI, WSXWS, and FVSVGPTYMRVS coupled to KLH), based on the published cDNA sequence of the 140-kD chain of the IL-4R (Galizzi et al., 1990), and fusion of immune spleen cells with NSO myeloma cells (Kearney et al., 1979). Immunization, fusion, HAT selection, and cloning were carried out as described previously (DeMaagd et al., 1985; Ladyman and Ritter, 1995). Screening was carried out by ELISA using immobilized individual peptides and by indirect immunoperoxidase staining of frozen tissue sections of human thymus.

MAb K21 was used as tissue-culture supernatant and its isotype determined using an isotyping kit (Serotec, Oxford, UK). Mouse anti-human CD3, CD4, CD5, CD7, CD14, CD19, CD20, CD22, CD23, CD45, and CD68 were also used as primary antibodies (all Dako, High Wycombe, UK). MAb MR6 (recognizes the gp200-MR6 molecule) is an IgG1 murine mAb and was used as tissue-culture supernatant (DeMaagd et al., 1985; Larché et al., 1988;

Imami et al., 1994). Second-layer reagents were horseradish peroxidase (HRP)-conjugated rabbit anti-mouse Ig, alkaline phosphatase (AP)-conjugated rabbit anti-mouse Ig, and fluorescein isothiocyanate (FITC)-conjugated rabbit anti-mouse Ig (all Dako). All antibodies were titrated before use.

### Immunohistochemistry

### Single indirect immunohistochemical staining of human thymus and tonsil sections

Tissue sections were incubated with primary antibody for 1 hr at room temperature, washed in PBS, and then incubated 1 hr further with either FITC- or HRP-conjugated rabbit anti-mouse Ig at 1/20 in PBS containing 5% normal human serum (NHS). For immunofluorescence, sections were mounted in a AF2 mountant (Citifluor, Canterbury, UK). For enzyme staining, sections were developed for 10 min using 6 mg diaminobenzidine (DAB; Sigma, Poole, UK) and 5 $\mu$l 30% $H_2O_2$ (Sigma) per 10 ml PBS, counter-stained with hematoxylin, and mounted in Kaiser's gelatin-based mountant. No staining was seen when the primary antibody was either omitted or replaced by an irrelevant isotype-matched control.

### Double immunoenzyme staining of human tonsil sections

Sections were incubated for 45 min with one set of primary antibodies, followed by HRP-conjugated secondary antibody (45 min), and developed as described before, and then incubated for an additional 45 min with the second set of primary and AP-conjugated rabbit anti-mouse Ig (45 min). AP labeling was developed over 20 min using 0.1% fast blue BB salt with 0.02% napthol-AS-MX phosphate, 0.2% levamisole (all Sigma), and 2% dimethyl formamide (BDH). All incubations were carried out at room temperature and all washes were with Tris-buffered saline (TBS), pH 7.6. No double staining was seen when either the first or the second primary mAb was omitted or replaced by the isotype-matched control.

### Biochemical Analysis of K21-Ag

For Western blotting analysis, 30 sections of frozen human tonsil tissue were cut on a cryostat at a thickness of 10 $\mu$m and lysed for 10 min in 1 ml of ice-cold lysis buffer (10 mM Tris/HCl, pH 7.4, containing 150 mM NaCl, 0.5% NP-40, and 1 mM PMSF; all Sigma) and then centrifuged in a 1.5-ml plastic tube (Eppendorf, Hamburg) at 13,000 g for 4 min at 4°C. The cell-free supernatant was mixed with an equal volume of either reducing (10% 2-ME or 300 mM DTT) or nonreducing double-strength Laemmli sample buffer, subjected to SDS-10%PAGE (Laemmli, 1970) and followed by electrophoretic transfer (300 mA for 1 to 1.5 hr; Towbin et al., 1979) onto nylon membranes (Milipore, Bedford, MA). Unoccupied charged sites on the membrane were blocked by immersion in 2.5% skimmed milk powder (Cadbury, Stafford, UK) in PBS for 3 to 24 hr. Membranes were cut into vertical strips that were incubated for 2 hr with either mAb K21 (1/3), anti-CD23, anti-CD45, anti-CD4 (all 1/10), mAb MR6 (1/3), or isotype-matched negative control mAb (H17E2; Travers and Bodmer, 1984) at ~10 $\mu$g/ml in 0.5% skimmed milk powder in PBS (this buffer was used for subsequent Ab dilutions and washing of strips). Following washing for 15 min in three changes of washing buffer, strips were incubated for 1 hr in a 1/100 dilution of HRP-conjugated rabbit anti-mouse Ig. Strips were then washed and peroxidase activity developed by incubation in 0.6 mg/ml DAB in 10 ml PBS, containing 5 $\mu$l $H_2O_2$.

### Indirect Immunofluorescence and Flow Cytometric Analysis: Surface and Internal Staining

For analysis by flow cytometry, $1 \times 10^6$ cells of each group were incubated with 100 $\mu$l (neat supernatant or 1/10 dilution of Dako mAbs) of the indicated mAb for 1 hr on ice in polystyrene round-bottomed tubes (Falcon, Becton-Dickinson, Lincoln Park, NJ) (Larché et al., 1988). After washing twice in medium, the cells were incubated for an additional 1 hr on ice with 100 $\mu$l of a 1/20 dilution of FITC-conjugated rabbit anti-mouse Ig. After three additional washes, stained cells were analyzed using an Epics XL-MCL, Flow cytofluorimeter with log amplifier (Coulter, USA). For internal staining, cells were treated with 0.1% saponin in PBS, and this buffer was used in all subsequent washes.

### Tonsillar Cell Separation and Mitogen Stimulation

T and B cells were separated by E-rosetting and nylon wool, respectively. For E-rosetting, cells were prepared by mixing tonsillar mononuclear cells with sheep red blood cells (SRBC; TCS, Buckingham, UK; modified from Callard et al., 1987). For nylon wool separation, tonsillar mononuclear cells were resuspended in 2 ml of RPMI medium, and loaded on a prewetted nylon wool (Biotest, Dreieich, Germany) column (0.2 g/2 ml column). The columns were then sealed and incubated at 37°C for 30 min, T cells were eluted by washing the columns twice with 10 ml warm (37°C) medium, while B cells were collected by applying 10 ml of ice-cold medium and agitating the wool vigorously. A sample of each of these cell preparations was stained by indirect immunofluorescence using both pan-B- and pan-T-cell markers (CD20, CD19, CD22 and CD3, CD5, CD7, respectively) to gauge their purity (using flow cytometry as described before). Tonsillar E-rosetted cells were routinely 80-90% ($CD3^+$) T cells, <10% ($CD20^+$) B cells. Tonsillar nylon-wool-bound cells were routinely 90-95% ($CD20^+$) B cells, <5% ($CD3^+$) T cells. In all experiments, 1% to 3% were monocytes ($CD14^+$, $CD68^+$). Separated B cells were cultured in both the presence and absence of lipopolysaccharides (LPS; 10 μg/ml; Sigma) for 24 hr, harvested, and analyzed by flow cytometry as described before.

### *Acknowledgements*

The authors would like to thank D. Gray for his helpful discussion, Sister Patricia Kelly at St. Mary's Hospital for collection of tonsil samples, and the staff at Great Ormond Street Hospital for Sick Children for collection of thymus samples.

### *References*

Al-Tubuly A.A., Luqmani Y.A., Shousha S., Melcher D., and Ritter M.A. (1996). Differential expression of gp200-MR6 molecule in benign hyperplasia and downregulation in invasive carcinoma of the breast. Brit. J. Cancer **74**: 1005-1011.

Bazan J.F. (1990). Structural design and molecular evolution of a cytokine receptor superfamily. Proc. Natl. Acad. Sci. USA **87**: 6934-6938.

Blau J.N. (1973). Hassall's corpuscles: A site of thymocyte death. Brit. J. Exp. Pathol. **54**: 634-637.

Boyd R.L., Tucek C.L., Godfrey D.I., Izon D.J., Wilson T.J., Davidson N.J., Bean A.G.D., Ladyman H.M., Ritter M.A., and Hugo P. (1993). The thymic microenvironment. Immunol. Today **14**: 445-459.

Callard R.E., Shields J.G., and Smith S.H. (1987). Assays for human B cell growth and differentiation factors. In Lymphokines and Interferons: A Practical Approach, Clemens M.J., Morris A.G., and Gearing A.J.H., Eds. (Oxford: I.R.L. Press), pp. 345-364.

DeMaagd R.A., Mackenzie W.A., Schuurman H.-J., Ritter M.A., Price K.M, Broekhuizen R., and Kater L. (1985). The human thymus microenvironment; heterogeneity detected by monoclonal anti-epithelial cell antibodies. Immunology **54**: 745-754.

Fough J. and Trempe G. (1975). New human tumour cell lines. In Human Tumour Cells In Vitro, Fogh J., Ed. (New York: Plenum), pp. 115-160.

Freysdóttir J., and Ritter M.A. (submitted). Influence of T cell-derived molecules on maintenance of cultured thymic epithelial cell.

Galizzi J.P., Zuber C.E., Harada N., Gorman D.M., Djossou O., Kastelein R., Banchereau J., Howard M., and Miyajima A. (1990). Molecular cloning of a cDNA encoding the human interleukin 4 receptor. Int. Immunol. **2**: 669-675.

Gray D. (1993). Immunological memory. Annu. Rev. Immunol. **11**: 49-77.

Imami N., Ladyman H.M., Spanopoulou E., and Ritter M.A. (1992). A novel adhesion molecule in the murine thymic microenvironment: Functional and biochemical analysis. Dev. Immunol. **2**: 161-173.

Imami N., Larché M., and Ritter M.A. (1994). Inhibition of alloreactivity by mAb MR6: Differential effects on IL-2- and IL-4-producing human T cells. Int. Immunol. **6**: 1575-1584.

Kampinga J., Berges S., Boyd R., Brekelmans P., Čolić M., van Ewijk W., Kendall M.D., Ladyman H., Nieuwenhuis P., Ritter M.A., Schuurman H.-J., and Tournefeier A. (1989). Thymic epithelial antibodies: Immunohistochemical analysis and introduction of nomenclature. Summary of the Epithelium Workshop held at the 2nd Workshop "The Thymus." Histophysiology and Dynamics in the Immune System. Thymus **13**: 165-173.

Kanariou M., Huby R., Ladyman H., Čolić M., Sivolapenko G., Lampert I., and Ritter M.A. (1989). Immunosuppression with cyclosporin A alters the thymic microenvironment. Immunology **78**: 263-270.

Kearney J.F., Radbruch A., Liesegang B., and Rajewsky K. (1979). A new mouse myeloma cell line that has lost immunoglobulin expression but permits the construction of antibody-screening hybrid cell line. J. Immunol. **123**: 1548-1550.

Ladyman H.M., and Ritter M.A. (1995). Production of monoclonal antibodies. In Monoclonal Antibodies, Ritter M.A., and Ladyman H.M., Eds. (Cambridge: Cambridge University Press), pp. 9-33.

Laemmli U.K. (1970). Cleavage of structural proteins during the assembly of the head of bacteriophage T4. Nature **227**: 680-685.

Larché M., Lamb J.R., O'Hehir R.E., Imami N., Zanders E.D., Quint D.E., Moqbel R., and Ritter M.A. (1988). Functional evidence for a monoclonal antibody that binds to the human IL-4 receptor. Immunology **65**: 617-622.

Laster A.J., Itoh T., Palker T.J., and Haynes B.F. (1986). The human thymic microenvironment: Thymic epithelium contains specific keratins associated with early and late stages of epidermal keratinocyte maturation. Differentiation **31**: 67-77.

Liberti E.A., Fagundes T.P., Perito M.A., Matson E., and Konig Jr. B. (1994). On the size of Hassall's corpuscles in human fetuses. Bull. Assoc. Anat. Nancy **78**: 15-18.

Liu Y-J., Cairns J.A., Holder M.J., Abbot S.D., Jansen K.U., Bonnefoy J-Y., Gordon J., and MacLennon I.C.M. (1991). Recombinant 25-KDa CD23 and interleukin 1a promote survival of germinal center B cells: Evidence for bifurcation in the development of centrocytes rescued from apoptosis. Eur. J. Immunol. **21**: 1107-1114.

MacLennan I.C.M. (1994). Germinal centers. Annu. Rev. Immunol. **12**: 117-139.

MacLennan I.C.M., Liu Y-J., and Johnson G.D. (1992). Maturation and dispersal of B cell clones during T cell-dependent antibody response. Immunol. Rev. **126**: 143-161.

Mat I., Melcher D., and Ritter M.A. (1991). Epithelial cell expression of interleukin-4 receptor complex. In Lymphatic Tissues in *In Vivo* Immune Responses, Ezine S., Berrih-Aknin S., and Imhof B., Eds. (New York: Marcel Dekker), pp. 27-32.

Mishalani S.H., Lones M.A., and Said J.W. (1995). Multilocular thymic cyst. A novel thymic lesion associated with human immunodeficiency virus infection. Arch. Pathol. Lab. Med. **119**: 467-470.

Miyajima A., Kitamura T., Harada N., Yokota T., and Arai K.-I. (1992). Cytokine receptors and signal transduction. Annu. Rev. Immunol. **10**: 295-331.

Numasaki M., Fukuoka Y., Kudo T., Saeki H., Tachibana T., Motomiya M., and Nukiwa T. (1995). A novel human monoclonal antibody, TONO-1, reactive with T-lymphocytic leukemia cells. Int. J. Cancer **62**: 42-47.

Palmer D.B., Viney J.I., Ritter M.A., Hayday A.C., Owen M.J. (1993). Expression of the $\alpha\beta$ T-cell receptor is necessary for generation of the thymic medulla. Dev. Immunol. **3**: 175-179.

Perry M.E., and Slipka J. (1993). Formation of the tonsillar corpuscle. Funct. Dev. Morphol. **3**: 165-168.

Peschon J.J., Morrissey P.J., Grabstein K.H., Ramsdell F.J., Maraskovsky E., Gliniak B.C., Park L.S., Ziegler S.F., Williams D.E., Ware C.B., Meyer J.D., and Davison B.L. (1994). Early lymphocyte expansion is severely impaired in interleukin 7 receptor-deficient mice. J. Exp. Med. **180**: 1955-1960.

Prevot S., Audouin J., Audre-Bougaran J., Griffais R., Le-Tourneau A., Fournier J.G., and Diebold J. (1992). Thymic pseudotumorous enlargment due to follicular hyperplasia in a human immunodeficiency virus sero-positive patient. Immunohistochemical and molecular biological study of viral infected cells. Amer. J. Clin. Pathol. **97**: 420-425.

Ritter M.A., and Boyd R.L. (1993). Development in the thymus: It takes two to tango. Immunol. Today **14**: 462-469.

Sharif M., Rook G., Wilkinson L.S., Worrall J.G., and Edwards J.C.W. (1990). Terminal N-acetylglucosamine in chronic synovitis. Brit. J. Rheumatol. **29**: 25-31.

Shikhman A.R., and Cunningham M.W. (1994). Immunological mimicry between N-acetyl-$\beta$-D-glucosamine and cytokeratin peptides. J. Immunol. **152**: 4375-4387.

Spencer J., Choy M., Hussell T., Papadaki L., Kington J.P., and Isaacson P.G. (1992). Properties of human thymic B cells. Immunology **75**: 596-600.

Sugamura K., Asao H., Kondo M., Tanaka N., Ishii N., Ohbo K., Nakamura M., and Takeshita T. (1996). The interleukin-2 receptor $\gamma$ chain: Its role in the multiple cytokine receptor complexes and T cell development in XSCID. Annu. Rev. Immunol. **14**: 179-205.

Towbin H., Staehelin T., and Gordon J. (1979). Electrophoretic transfer of proteins from polyacrylamide gels to nitrocellulose sheets: Procedure and some applications. Proc. Nat. Acad. Sci. USA **76**: 4350-4354.

Travers P., and Bodmer W.F. (1984). Preparation and characterisation of monoclonal antibodies against placental alkaline phosphatase and other human trophoblast-associated determinants. Int. J. Cancer **33**: 633-641.

Van Ewijk W. (1991). T-cell differentiation is influenced by thymic microenvironments. Annu. Rev. Immunol. **9**: 591-615.

Zlotnik A., and Moore T.A. (1995). Cytokine production and requirements during T-cell development. Curr. Opin. Immunol. **7**: 206-213.

